# Field performance of *Plasmodium falciparum* lactate dehydrogenase rapid diagnostic tests during a large histidine-rich protein 2 deletion survey in Ethiopia

**DOI:** 10.1186/s12936-022-04257-9

**Published:** 2022-08-15

**Authors:** Sindew Mekasha Feleke, Bokretsion Gidey, Hussein Mohammed, Desalegn Nega, Dereje Dillu, Mebrhatom Haile, Hiwot Solomon, Jonathan B. Parr, Getachew Tollera, Geremew Tasew, Hassen Mamo, Beyene Petros

**Affiliations:** 1 Ethiopian Public Health Institute, Addis Ababa, Ethiopia; 2 Ministry of Health, Addis Ababa, Ethiopia; 3 Department of Microbial, Cellular and Molecular Biology, College of Natural and Computational Sciences, Addis Ababa University, Addis Ababa, Ethiopia; 4 Institute for Global Health and Infectious Diseases and Department of Medicine, Division of Infectious Diseases, University of North Carolina at Chapel Hill (UNC), Chapel Hill, NC USA

**Keywords:** Malaria, PLDH, Diagnostics, Elimination, Ethiopia

## Abstract

**Background:**

Malaria rapid diagnostic tests (RDTs) have expanded diagnostic service to remote endemic communities in Ethiopia, where 70% of malaria services per annum are reliant on them. However, diagnostic strategies are threatened by *Plasmodium falciparum* parasites with deletions of the histidine-rich protein 2 and/or 3 (*pfhrp2/3*) genes. Studies have reported *pfhrp2/3* gene deletion prevalence in Ethiopia that exceeds the WHO recommended threshold to switch to non-HRP2 targeted RDTs for detection of *P. falciparum*. Therefore, RDTs that target alternative antigens, such as *P. falciparum* lactate dehydrogenase (PfLDH) are increasingly in programmatic use.

**Methods:**

Malaria suspected patients visiting health facilities of Amhara, Tigray, Gambella, and Oromia regions of Ethiopia were screened by community health workers using Carestart Pf/Pv* (*HRP2/Pv-LDH*)* and SD-Bioline Pf* (*HRP2 for Pf/LDH for Pf*)* RDTs. Dried blood spot (DBS) samples were collected from selected patients for molecular and serological analysis. The clinical data and RDT results were recorded on standard forms, entered into EpiInfo, and analysed using STATA. The Pf-LDH detecting RDT results were compared with real-time PCR and bead-based immunoassay to determine their diagnostic performance.

**Results:**

The 13,172 (56% male and 44% female, median age of 19 years ranging from 1 to 99 year) study participants were enrolled and tested with PfHRP2 and PfLDH detection RDTs; 20.6% (95% CI: 19.6 to 21.6) were *P. falciparum* RDT positive. A subset of samples (n = 820) were previously tested using *P. falciparum* lactate dehydrogenase (*pfldh*) quantitative real-time PCR, and 456 of these further characterized using bead-based immunoassay. The proportion of samples positive for *P. falciparum* by the PfHRP2 Carestart and SD-Bioline RDTs were 66% (539/820) and 59% (481/820), respectively; 68% (561/820) were positive for the PfLDH band on the SD-Bioline RDT. The sensitivity and specificity of the PfLDH RDT band were 69% and 38%, respectively, versus *pfldh* qPCR; and 72% and 36%, respectively, versus PfLDH detection by immunoassay. Among samples with results for RDT, qPCR, and immunoassay, higher proportions of *P. falciparum* were recorded by *pfldh* qPCR (90%, 411/456) and PfLDH immunoassay (88%, 363/413) compared to the PfLDH band on the SD-Bioline RDT (74.6%, 340/456).

**Conclusion and recommendation:**

Both PfHRP2 RDTs detected fewer *P. falciparum* cases than PfLDH, and fewer cases than qPCR or immunoassay. The poor sensitivity and specificity of the PfLDH RDT compared to qPCR and to immunoassay in this study raises concern. Continuous operator training and RDTs quality assurance programme to ensure quality diagnostic services are recommended.

## Background

Human malaria is a mosquito-borne parasitic disease caused by protozoan parasites belonging to the genus *Plasmodium* [1]. *Plasmodium falciparum, Plasmodium vivax, Plasmodium malariae, Plasmodium ovale* and *Plasmodium knowlesi* are the aetiologic agents of human malaria, of which *P. falciparum* is responsible for the most severe forms of the disease [2]. Over the last decade, there has been a tremendous reduction of malaria cases and deaths worldwide. Accordingly, mortality has reduced by 60% between 2000 and 2019 worldwide, whereas in Africa, where 94% of cases are accounted for, the annual mortality has fallen from 680,000 in 2000 to about 384,000 in 2019 [2].

In Ethiopia, malaria is one of the most important public health problems with more than 60% of Ethiopians at risk [3]. The country developed an elimination roadmap in 2016, which aims to reduce malaria incidence and mortality to zero by 2030 and prevent the re-establishment of malaria in all malaria-free areas [3]). Thus, the national programme is intensively implementing the elimination strategies, including parasitological diagnosis using rapid diagnostic tests (RDTs) or microscopy, and prompt treatment and prevention of relapse, using safe and effective anti-malarial drugs, vector control measures using indoor residual spray (IRS) and long-lasting insecticidal nets (LLINs), and health education.

Malaria case management is a crucial elimination strategy to reduce morbidity, mortality, prevent subsequent transmission and manage non-malarial febrile illnesses [4]. Diagnostic service provision in remote areas has been possible through the wide use of RDTs, an antigen detection test that gives rapid results with minimal operator training [5]. Malaria RDTs can detect the following three reliable target antigens: histidine-rich protein-2 (HRP2*),* parasite-specific lactate dehydrogenase (LDH) (species specific and pan specific)*,* and *Plasmodium* aldolase, of which HRP2 antigen is the preferred and most commonly used for the detection of *P. falciparum,* due to its high abundance in the blood, high heat stability and species-specificity [6]. In sub-Saharan Africa, 99% of malaria is caused by *P. falciparum* [1]. Whereas in Ethiopia, 60–70% of annual malaria is caused by *P. falciparum, *30–40% of cases are caused by *P. vivax,* and < 1% by both *P. ovale* and *P. malariae* based on routine facility report [3]. The small-scale community-based sero survey in Ethiopia has shown up to 11% *P. ovale* and 7% *P*. *malariae* antibody exposure history [7]. A study in China indicated that 1.5% confirmed *P. ovale curtisi* cases imported from Ethiopia [8].

Light microscopy is the gold standard for diagnosis of clinical malaria [9]. However, malaria RDTs are widely used in Ethiopia particularly in health posts by community health workers also called health extension workers (HEWs) and in health facilities that have no electricity, which accounts for about 70% of malaria diagnostic services per annum [3]. The national programme is using HRP2 and LDH detecting combination RDTs targeting *P. falciparum* and *P. vivax,* respectively [3], with detection capacity limited to both species only but not the *P. ovale* and *P*. *malariae* parasites. Besides this limitation, the malaria diagnostic programme is threatened by the emergence and spread of *P. falciparum* parasites that have single or dual deletion mutations of the *pfhrp2/3* genes and hence not detected by the widely used HRP2*-*detecting RDTs [10].

A recent large survey conducted in three regions of north and west Ethiopia and estimated that HRP2-based RDTs would miss 9.7% of symptomatic falciparum malaria cases owing to deletion of the *P. falciparum* histidine-rich protein 2 (*hrp2*) genes [11]*.* Because this estimate exceeds the WHO-recommended 5% prevalence threshold for transitioning to non-HRP2 detecting RDTs, the national malaria programme is evaluating RDTs that detect alternative antigens for programmatic use. The only available alternatives *P. falciparum-*specific RDTs that meet procurement criteria detect *P. falciparum-*specific parasite lactate dehydrogenase (PfLDH) [12]. In the initial paper, the prevalence of false-negative RDTs in the three regions tested and the genetic epidemiology of parasites with *pfhrp2* and/or *pfhrp3* gene deletions has been described. In this paper, the analysis includes the performance of two WHO pre-qualified RDTs, to include comparison to bead-based immunoassay results for HRP2, aldolase, and parasite LDH antigens and to include additional samples collected from the Oromia region that were not included in the original study.

## Methods

### Study site and design

**Fig. 1:**
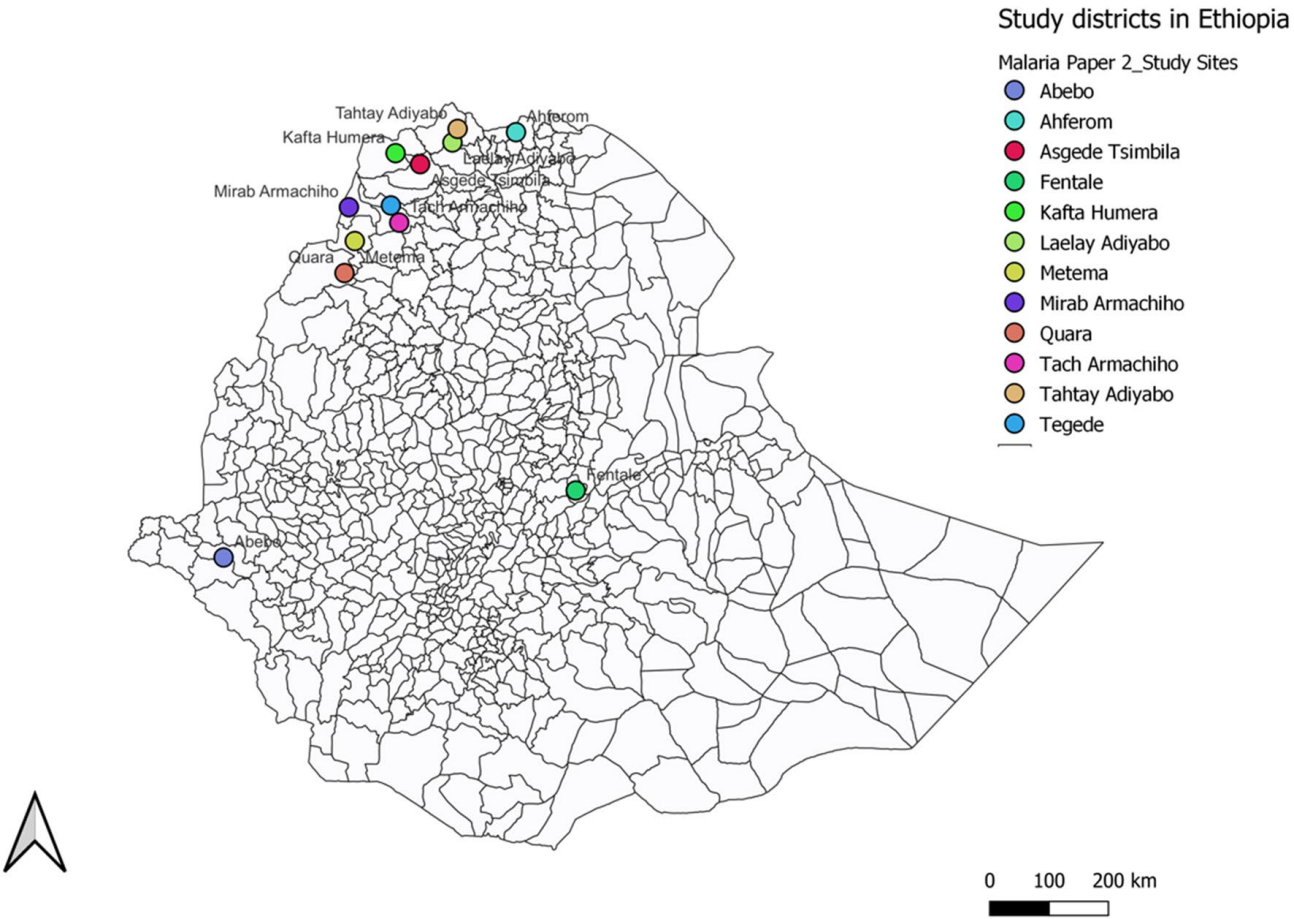
Study districts in Amhara, Tigray, Oromia and Gambella regions, Ethopia

This cross-sectional survey was conducted in selected health facilities of Amhara, Tigray, and Gambella regions of Ethiopia (Fig. 1) between 2017and 2018 as described in previous study [11]. The present study also includes subjects from the Oromia region who were enrolled following the same procedures exactly as described in the previous work [11]. In brief, malaria suspected patients visiting the health facilities were enrolled in the study, and each patient was subjected to laboratory tests using Care Start Pf/Pv (HRP2/LDH*)* RDT (product code RM 103 VM-02571) and SD Bioline Malaria Ag Pf (HRP2 for Pf/LDH for Pf) (product code 05FK90). The RDTs were performed using finger-prick fresh whole blood following the manufacturer's instructions. Dried blood spot (DBS) samples were collected from selected positive patients for advanced testing run based on the published standard protocols for immunoassay [13] at the Centers for Disease Prevention and Control (CDC) laboratory, Atlanta, USA, and PCR assays [14] in North Carolina University, Chapel Hill, USA. The qPCR test is used to confirm the *Plasmodium* species, quantify the parasite and test *P. falciparum (hrp2, pfldh* and *aldolase*) target genes. Whereas, the bead based immunoassay [7] was used to test for HRP2, LDH and Aldolase antigens in the samples.

All enrolled subjects provided informed consent, and any subject with a positive test for malaria received treatment as directed by national guidelines. The study was carried out after the ethical approval is obtained from the Ethiopia Public Health Institute Institutional Review Board (IRB; protocol number EPHI-IRB-033–2017 for Amhara, Tigray and Gambella study, and EPHI-6.13/596 28 Jan 2020 for Oromia studies) and the WHO Ethics Review Committee Protocol number: ERC.0003174. Data cleaning and analysis was performed using Stata SE version 14 software. The association of the clinical and demographic variables with the *P. falciparum* positivity was measured using the T-test. The sensitivity and specificity of PfHRP2 and PfLDH detecting RDTs (screening tests) were performed against its qPCR and immunoassay (reference tests) results.

## Results

A total of 13,172 malaria-suspected patients, 56% of whom were male and 44% female with an age range between 0 and 99 years and median age of 19 years who have visited health facilities in Amhara (30% study participants), Tigray (50%), Oromia (4%), and Gambella regions (16%) were enrolled in the study. Of all malaria suspected patients, 20.6% (2714/13172) were *P. falciparum* positives either by one or both RDTs. The patient’s information on clinical sign and symptoms showed a 91.4% fever cases (reported), 63.5% joint pain, 58% poor appetite, 57.4% feeling cold, 45% nausea and 18.4% headache. In addition, 75.7% were rural residents and, 6% (n = 777) of them had history of anti-malarial treatment a month before enrolled in the study, of which 31% received ACT, 11% chloroquine, 7% quinine, and 51% other drugs. All the variables including sex, clinical signs and symptoms, house location and anti-malarial treatment history had strong association with *P. falciparum* positivity (P < 0.001) (Table 1).

**Table 1:** T-test P-values evaluate differences in *P. falciparum* malaria (outcome) for each variable (exposure)

Variables	Category	Frequency, n (%)	RDT *Pf cases*, n (%)	*P-value*
Sex	Female	5810 (44)	1823 (67.2)	< 0.001
	Male	7363 (56)	891(32.8)	
House location	Urban	3124 (24)	458 (16.9)	< 0.001
	Rural	8169 (62)	2055 (75.7)	
Fever	Yes	12,043 (91.4)	2620 (96.5)	< 0.001
	No	1128 (8.6)	93 (3.4)	
Headache	Yes	10,741 (18.4)	2535 (93.4)	< 0.001
	No	2430 (81.5)	178 (6.6)	
Joint pain	Yes	8371 (63.5)	2166 (79.8)	< 0.001
	No	4801 (36.4)	547 (20.2)	
Feeling cold	Yes	7565 (57.4)	1826 (67.3)	< 0.001
	No	5607 (42.6	887 (32.7)	
Nausea	Yes	5933 (45)	1522 (56)	< 0.001
	No	7219 (55)	1186 (44)	
Poor appetite	Yes	7647 (58)	2022 (75)	< 0.001
	No	5482 (42)	686 (25)	
Treatment history	Yes	777 (6)	272 (10)	< 0.001
	No	12,395 (94)	2440 (90)	
	Total (n)	13,172	2714 (20.6%)	

Molecular analysis was carried out in selected samples (n = 820) from *P. falciparum* cohort samples. The proportion of *P. falciparum* in PfHRP2 detecting CareStart RDT and SD-Bioline RDT were 66% (539/820) and 58.7% (481/820), respectively (Table 2). Whereas, the PfLDH targeted RDTs and *pfldh* PCR result showed 68% (561/820) and 86% (701/811) *P. falciparum*, respectively (Table 2). The PfHRP2 and PfLDH RDT test results showed a 97% high agreement (k: 0.94).

**Table 2:** Comparison of Pf RDT results with *PfLDH* PCR detection assay

Test type	Proportion of target antigen or gene positive samples, n (%)					Total (n)
	HRP2, n/%	PvPLDH, n/%	PfPLDH n/%	*pfldh,* n/%	Ng, n/%	
CareStart Pf/Pv	539 (66)	88 (11)	*		245 (30)	820
SD Pf	481(58.7)	*	561(68)		70 (8.5)	820
*pfldh* PCR	*	*	*	701(86)	110 (14)	811

Key: * = not applicable, *n* number of samples, *%* percentage, *Ng* negative

The sensitivity and specificity of the PfLDH targeted RDT result compared with the *pfldh* PCR assay was 69% and 38%, respectively, as previously reported [11] (Table 3). Here the false-negatives further analysed showed most (29%) of them had low parasitaemia (< 100 p/ul); low-density infections may have antigenaemia below the detection threshold of the RDTs. If the 36% (83/225) low-parasitaemia (< 200 p/µl) false-negatives are excluded from the analysis, given that they are below the expected RDT limit of detection, the PfLDH RDT’s sensitivity and specificity improve to 78% and 68%, respectively. However, 23% false negatives had > 2000 p/µl – well above the RDTs limit of detection – suggestive of operator, procedural, product related or administrative errors during RDT use (Table 3).

**Table 3:** PfLDH RDT’s sensitivity & the parasitaemia among RDT false-negatives* (n = 225)

	pfldh PCR			
PfLDH RDTs	Pos	Ng	Total	Sensitivity = 69%
Pos	506	55	561	PPV = 90.2
Ng	225*	34	259	Specificity = 38%
Total	731	89	820	

Parasitaemia (p/µl)	Number (%)
< 100 (range 3–95 p/µl)	66 (29)
100–200	17 (8)
200–500	33 (15)
500–1000	29 (13)
1000–2000	28 (12)
> 2000	52 (23)
Total	225* (100)

Key: * = RDT false-negative samples

*Ng * negative, *Pos* positive

A total of 225 *pfldh* PCR positives were not detected by PfLDH RDTs

In addition, 456 of these 820 samples were further tested using bead-based immunoassay. The results in PfHRP2 detection by CareStart RDT, SD-Bioline RDT, PCR and bead-based immunoassay showed 61% (278/456), 55% (251/456), 57% (260/456) and 65% (297/456) *P. falciparum* positivity, respectively (Table 4). Whereas, the PfLDH-detecting SD Bioline RDT, *pfldh* PCR and PfLDH bead-based immunoassay showed 74.6% (340/456), 90% (411/456) and 88% (363/413) positives, respectively (Table 4). The PfLDH-targeted RDTs results compared with PLDH bead based immunoassay showed 72% and 36% sensitivity and specificity, respectively.

**Table 4:** Comparison of *P. falciparum* RDT results with PCR and bead based multiplex immunoassay (BBMI)

Test type	Proportion of target antigen or gene positives, n (%)						Total (n)
	HRP2, n (%)	PvPLDH, n (%)	PfPLDH, n (%)	Aldolase, n (%)	*pfldh,* n (%)	Ng, n (%)	
CareStart Pf/Pv	278 (61)	15 (3.3)	*	*		164 (36)	456
SD Pf	251 (55)	*	340 (74.6)	*		28 (6)	456
*pfldh* PCR	*	*	*	*	411 (90)	45 (10)	456
BBMI (HRP2)	297 (65)	*	*	*		159 (35)	456
BBMI (PfLDH)	*	*	363 (88)	*		50 (12)	413
BBMI (Aldolase)	*	*	*	272(66)		141 (34)	413

Key: * =not applicable, *n* = number of samples, *%* percentage, *Ng* negative

## Discussion

Malaria case management through accurate diagnosis and prompt treatment is vital especially in elimination setting. In this study, relatively low malaria cases (21%) in spite of high fever prevalence (91%) in 13,172 self-presenting malaria suspected patients are reported which implies the need for accurate parasitological confirmation of cases for the rational use of treatment and management of non-malaria illnesses [15]. Given *P. falciparum* is the major cause of malaria mortality and morbidity in Africa, effective case management is needed to reach the goal of elimination. The use of malaria rapid diagnostic tests for the national diagnostic programme since the 1990^th^ has been a backbone with significant increase of use over the last two decades which accounts for over 70% of diagnosis in Ethiopia [3]. However, the emergence of *pfhrp2/3* gene mutated parasites in Ethiopia [7] is threatening because the widely used PfHRP2-detecting RDT is compromised. A recent study in Ethiopia reported high prevalence (~ 10%) of *pfhrp2/3* gene deletions causing false negative HRP2-RDT results, which is above the 5% WHO prevalence threshold to change the diagnostic strategy [16], as a result the country is shifting to non-HRP2 (i.e. PfLDH) detection RDTs for programme use.

This study evaluates the performance of PfLDH detection RDT for programme use and presents the proportion and sensitivity of *P. falciparum* in HRP2 and PfLDH targeted RDTs, qPCR and multiplex assay results. The results show that generally higher proportion of *P. falciparum* is reported by the PfLDH RDT, *pfldh* qPCR and PfLDH multiplex assays (74%, 90% and 88%, respectively), with more cases are identified by PCR and bead-based immunoassay, compared to HRP2 RDTs and HRP2 bead-based immunoassay (55% and 65%, respectively) which implies that the PfLDH target antigen is performing better than HRP2 antigen detection for *P. falciparum* prevalence estimation.

Both RDTs used during the survey are WHO prequalified and, therefore, should meet requirements for safety and quality ([17] and https://extranet.who.int/pqweb/vitro-diagnostics/vitro-diagnostics-lists). However, the performance of malaria RDT is dependent on several factors in addition to parasite densities [18] such as reduced community level transmission intensities [19]; operator skill [20, 21], correct transport and storage conditions [22] and a strong product lot quality monitoring program [21]. In spite of high performance of PfLDH detection RDTs reported by others [12, 23–25], the lower sensitivity and specificity of LDH detection RDT reported in this study, 69% and 72% sensitivity against PCR and multiplex assay, respectively and 38% and 36% specificity against PCR multiplex, respectively, could be partly driven by samples with low parasitaemia below the detection threshold of the PfLDH RDT. Indeed, 29% of LDH detection RDT false-negatives are below 100 p/µl. In addition, operator’s skill gaps and low intensities of the transmission as most of this study sites are in elimination phase with annual parasite incidence (API) of less than one per 1000 population [7]. Therefore, laboratory and field level quality monitoring, operator’s refresher training, storage and transportation temperature monitoring are important to improve malaria RDTs service quality.

## Limitation of the study

Subjects were enrolled using an adaptation of the WHO protocol for *pfhrp2/3* deletion surveillance that prioritized enrollment of participants with discordant RDT results (16). This study design could introduce selection bias that limits the generalizability of our findings to the broader population. Nonetheless, analysis of multiple diagnostic assays applied to each sample provides a valuable opportunity to evaluate the performance of PfLDH RDTs. In addition, the field-level RDT tests were carried out by community health workers in remote health posts which could have impacted the results related to operators limited skills, inaccessibility for adequate supervision and, uncontrolled storage and transportation conditions. For example, faint test bands are not uncommon with the PfLDH-detecting RDT evaluated here and might be detected by expert readers, but missed by community health workers.

## Conclusion and recommendation

The field-level performance of PfLDH detection RDTs results showed that the overall proportion of *P. falciparum* cases detected by PfLDH detecting RDT, *pfldh* qPCR and a PfLDH immunoassay is generally higher than HRP2 detection by RDT or immunoassay. However, the sensitivity and specificity of PfLDH detection RDTs compared to both *pfldh* PCR and to PfLDH bead-based immunoassay was lower than expected. As the country is preparing to switch to non-HRP2 detection in response to *pfhrp2* deletion problem, a strong RDT quality assurance system and adequate in-service training for operators is needed before introducing alternative RDT products for national programme use.

## Data Availability

The database analysed in this study are available from the corresponding author.
